# Characterization of the complete chloroplast genome sequence of *Sarcandra glabra* (Chloranthales)

**DOI:** 10.1080/23802359.2020.1715858

**Published:** 2020-01-24

**Authors:** Wei Wang, Peishan Zou, Guofeng Liu, Seping Dai

**Affiliations:** Guangzhou Institute of Forestry and Landscape Architecture, Guangzhou, China

**Keywords:** *Sarcandra glabra*, chloroplast genome, phylogenetic analysis

## Abstract

*Sarcandra glabra* is a perennial evergreen subshrub, with high ornamental and medicinal value. Using the Illumina high-throughput sequencing data, its chloroplast genome is well assembled and characterized. The complete chloroplast genome is 158,872 bp in length with a typical quadripartite structure: a pair of inverted repeats (IRs) of 26,122 bp for each, an 88,182 bp large single-copy (LSC) region and an 18,445 bp small single-copy (SSC) region. It was composed of 128 genes and they were identified 84 coding genes, 8 rRNA genes, 36 tRNA genes. Phylogenetic analysis confirmed that the position of *S. glabra* lay within the order Chloranthales instead of Piperales simply according to classical morphological taxonomy.

*Sarcandra glabra* (Thumb.) Nakai, a perennial evergreen subshrub, phylogenetically belongs to Chloranthales according to APG IV (Angiosperm Phylogeny Group et al. 2016). It has a wide range of uses: its leaves can extract aromatic oil (Wong et al. 2009); its spikes and red globular drupe endow it of high ornamental value in horticulture; the whole plant of it can process into a kind of Chinese traditional herbal medicine, which has anti-bacterial and anti-inflammatory effect (Tsai et al. 2017); its fragrance in all season, which benefits to people’s physical and mental health, makes it an ideal indoor potted planting.

At present, the research on *S. glabra* mainly focuses on its morphological characteristics, pharmacology and clinical application (Pan et al. 2004; Xu et al. 2008), cultivation and reproduction technology (Maria and Peter 1999; Tosaki et al. 2001) and genetic diversity of germplasm resources (Ni et al. 2008; Tang et al. 2012; Wei et al. 2014). The acquisition of the chloroplast genome can be a good supplement for the scarce genomic resources of this species, when regarding conservation concerns, sustainable utilization and taxonomy of this species. Therefore, we sequenced and characterized the complete chloroplast genome of *S. glabra*.

A strain of *S. glabra* was sampled from Guangzhou Institute of Forestry and Landscape Architecture. Genomic DNA was extracted from mature leaves in good condition using a modified CTAB method (Doyle and Doyle 1987), then purified to construct a 150 bp DNA library before sequencing on an Illumina Hiseq X10 platform. The residual whole plant was processed to a voucher specimen (specimen code SYS-Bore-2018-07-18), deposited in Sun Yat-sen University Herbarium. We finally got 4.42 Gbp paired-end clean data with 93.82% ≥ Q30, which was used to launch an assembly of complete chloroplast genome together with *rbc*L gene sequence (GenBank accession No. MH270458.1) as a seed on a Perl script, NOVOPlasty (Dierckxsens et al. 2017). Online software DOGMA (Wyman et al. 2004) was used to automatically annotate the chloroplast genome, followed by manual double-check and adjustment. We then used OGDRAW (Lohse et al. 2013) to visual the gene map of the *S. glabra* chloroplast genome. The Genbank accession of the complete chloroplastic sequence was MH939147.1.

It was a typical quadripartite circular form with 158,872 bp in length and comprised a large single copy (LSC, 88,182 bp) region, a small single copy (SSC, 18,445 bp) region, and two inverted repeat (IR, 26,122 bp) regions. It was composed of 128 genes and 84 coding genes, 8 rRNA genes, 36 tRNA genes were identified.

In the phylogenetic tree, the polyphyly between Chloranthales (including *S. glabra*) and Piperales was strongly supported (Figure 1).

**Figure 1.**
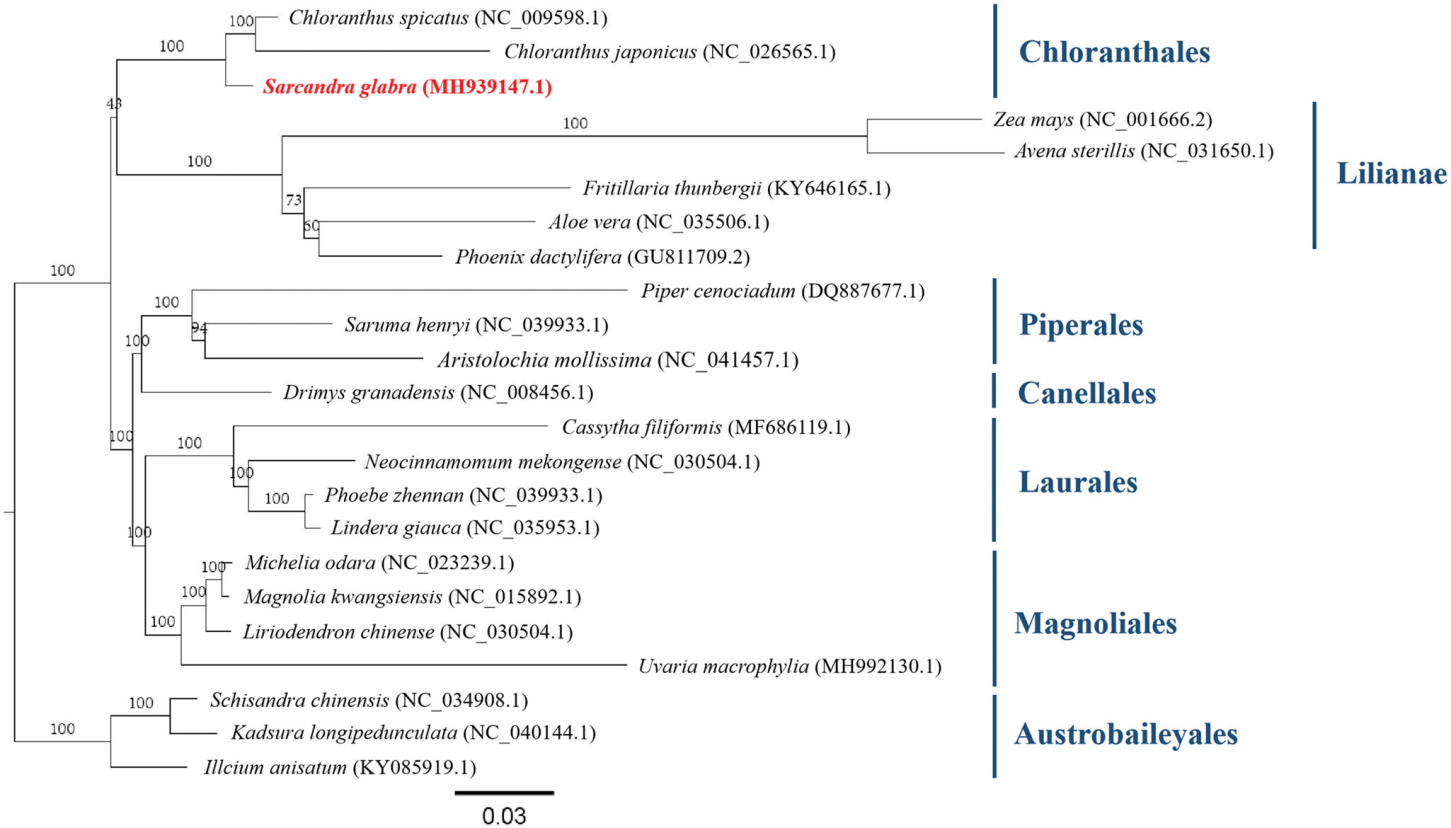
Maximum likelihood tree showing the phylogenetic position of *Sarcandra glabra* based on the complete chloroplast genome sequences. Bootstrap support values (1000 replicates) are shown next to the nodes. Scale in substitutions per site.

## References

[CIT0001] Angiosperm Phylogeny Group, Chase MW, Christenhusz MJM, Fay MF, Byng JW, Judd WS, Soltis DE, Mabberley DJ, Sennikov AN, Soltis PS, et al. 2016. An update of the Angiosperm Phylogeny Group classification for the orders and families of flowering plants: APG IV. Bot J Linn Soc. 181(1):1–20.

[CIT0002] Dierckxsens N, Mardulyn P, Smits G. 2017. NOVOPlasty: *de novo* assembly of organelle genomes from whole genome data. Nucleic Acids Res. 45(4):e18. 28204566 10.1093/nar/gkw955 PMC5389512

[CIT0003] Doyle JJ, Doyle JL. 1987. A rapid DNA isolation procedure for small quantities of fresh leaf tissue. Phytochem Bull. 19:11–15.

[CIT0004] Lohse M, Drechsel O, Kahlau S, Bock R. 2013. OrganellarGenomeDRAW – a suite of tools for generating physical maps of plastid and mitochondrial genomes and visualizing expression data sets. Nucleic Acids Res. 41:575–581. 23609545 10.1093/nar/gkt289 PMC3692101

[CIT0005] Maria B, Peter E. 1999. Floral bract function, flowering process and breeding systems of *Sarcandra* and *Chloranthus* (Chloranthaceae). Plant Syst Evol. 218(3/4):161–178.

[CIT0006] Ni K, Fang M, Guo E, Si J, Zhang Z, Li H. 2008. Analysis on genetic diversity of *Sarcandra glabra* collected from eight provenance based on ISSR markers. Chin Trad Herb Drug. 39:1392–1396.

[CIT0007] Pan C, Xu H, Peng H, Ou W, Lu S. 2004. [Resource investigation and exploitable foreground of *Sarcandra glabra*]. J Chin Med Mater. 27(8):556–557 (Chinese). 15658813

[CIT0008] Tang M, Wei R, Yao S, Lan Z, Ling Z. 2012. [AFLP analysis on genetic diversity of *Sarcandra glabra* in Guangxi region]. Chin Trad Herb Drugs. 43(7):1398–1420 (Chinese).

[CIT0009] Tosaki Y, Renner SS, Takahashi H. 2001. Pollination of *Sarcandra glabra* (Chloranthaceae) in natural populations in Japan. J Plant Res. 114(4):423–427.

[CIT0010] Tsai Y, Chen S, Lin L, Fu S. 2017. Anti-inflammatory principles from *Sarcandra glabra*. J Agric Food Chem. 65(31):6497–6505. 28110531 10.1021/acs.jafc.6b05125

[CIT0011] Wei Y, Chen Y, Luo L, Yang Q, Chen Y, Liang Y. 2014. [Genetic relationship and parent selection of some *Sarcandra glabra* resources based on ISSR]. China J Chin Mater Med. 39(23):4571–4575. Chinese. 25911803

[CIT0012] Wong K, Tan M, Ali DMH, Teoh SG, Osman H, Tan S. 2009. Essential oil of the leaves of *Sarcandra glabra* (Thunb.) Nakai. J Essent Oil Res. 21(1):71–73.

[CIT0013] Wyman SK, Jansen RK, Boore JL. 2004. Automatic annotation of organellar genomes with DOGMA. Bioinformatics. 20(17):3252–3255. 15180927 10.1093/bioinformatics/bth352

[CIT0014] Xu X, Hu X, Yuan J, Yang J. 2008. [Studies on chemical constituents of *Sarcandra glabra*]. China J Chin Mater Med. 33(8):900–902 (Chinese). 18619347

